# Age‐associated cholesterol reduction triggers brain insulin resistance by facilitating ligand‐independent receptor activation and pathway desensitization

**DOI:** 10.1111/acel.12932

**Published:** 2019-03-18

**Authors:** Adrián Martín‐Segura, Tariq Ahmed, Álvaro Casadomé‐Perales, Irene Palomares‐Perez, Ernest Palomer, Axelle Kerstens, Sebastian Munck, Detlef Balschun, Carlos G. Dotti

**Affiliations:** ^1^ Department of Molecular Neuropathology, Centro de Biología Molecular Severo Ochoa CSIC/UAM Madrid Spain; ^2^ Department of Developmental and Molecular Biology Albert Einstein College of Medicine Bronx New York; ^3^ Faculty of Psychology & Educational Sciences University of Leuven Leuven Belgium; ^4^ Neurological Disorders Research Center QBRI‐HBKU Doha Qatar; ^5^ Cell & Developmental Biology Department University College London London UK; ^6^ Department of Neuroscience, VIB Center for Brain and Disease Research University of Leuven Leuven Belgium

**Keywords:** aging, cholesterol, insulin signaling

## Abstract

In the brain, insulin plays an important role in cognitive processes. During aging, these faculties decline, as does insulin signaling. The mechanism behind this last phenomenon is unclear. In recent studies, we reported that the mild and gradual loss of cholesterol in the synaptic fraction of hippocampal neurons during aging leads to a decrease in synaptic plasticity evoked by glutamate receptor activation and also by receptor tyrosine kinase (RTK) signaling. As insulin and insulin growth factor activity are dependent on tyrosine kinase receptors, we investigated whether the constitutive loss of brain cholesterol is also involved in the decay of insulin function with age. Using long‐term depression (LTD) induced by application of insulin to hippocampal slices as a read‐out, we found that the decline in insulin function during aging could be monitored as a progressive impairment of insulin‐LTD. The application of a cholesterol inclusion complex, which donates cholesterol to the membrane and increases membrane cholesterol levels, rescued the insulin signaling deficit and insulin‐LTD. In contrast, extraction of cholesterol from hippocampal neurons of adult mice produced the opposite effect. Furthermore, in vivo inhibition of Cyp46A1, an enzyme involved in brain cholesterol loss with age, improved insulin signaling. Fluorescence resonance energy transfer (FRET) experiments pointed to a change in receptor conformation by reduced membrane cholesterol, favoring ligand‐independent autophosphorylation. Together, these results indicate that changes in membrane fluidity of brain cells during aging play a key role in the decay of synaptic plasticity and cognition that occurs at this late stage of life.

## 1 INTRODUCTION

Brain insulin has been traditionally associated with important roles in the organism: regulation of appetite, body temperature, response to hypoglycemia, and others (Kleinridders, Ferris, Cai, & Kahn, 2014). In addition, recent studies strongly indicate a central role for insulin in cognitive processes and the underlying synaptic processes (Biessels & Reagan, 2015; Haj‐ali, Mohaddes, & Babri, 2009; Moosavi, Naghdy, & Choopani, 2007). Thus, insulin modulates long‐term potentiation (LTP) in the hippocampal CA1 region (Grillo et al., 2015; Martin et al., 2012; Nisticó et al., 2012) and can induce on its own, that is, without the need of further electrical stimuli, hippocampal long‐term depression (LTD; hereinafter referred to as insulin‐LTD; Ahmadian et al., 2004; Huang, Lee, & Hsu, 2004; Man et al., 2000). LTD is increasingly recognized as a second main form of synaptic plasticity that is important for learning and memory (Collingridge, Peineau, Howland, & Wang, 2010; Ge et al., 2010; Kemp & Manahan‐Vaughan, 2007). This also provides, at least in part, the rationale for the role of brain insulin in the development of neurodegenerative diseases and the clinical impact of intranasal insulin administration on mood and memory recall (Benedict et al., 2007; Carro, Trejo, Gomez‐Isla, LeRoith, & Torres‐Aleman, 2002; Freiherr et al., 2013).

Aging affects insulin and insulin growth factor 1 (IGF‐1) signaling (Fernandes, Saad, & Velloso, 2001; Frölich et al., 1998). Clinical and preclinical studies have shown a reduction of insulin and IGF‐1 receptors, their message, and their function in the hippocampus during normal and pathological aging (Deak & Sonntag, 2012; Talbot & Wang, 2014; Zaia & Piantanelli, 2000). Altered brain insulin signaling in the old often accompanies peripheral insulin resistance due to metabolic deregulation in type 2 diabetes (T2D; Biessels & Reagan, 2015; Bruehl et al., 2009) or neurodegenerative diseases such as Alzheimer's disease (AD; Talbot et al., 2012). However, brain insulin resistance also develops independently of T2D or AD (Steculorum et al., 2014) and it remains unclear how normal aging can induce these alterations.

Here, we propose that the brain insulin signaling deficit with age is the consequence of the changes in the lipid composition of the neuronal plasma membrane that occurs with natural aging (Martín, Pfrieger, & Dotti, 2014). We envision that changes in lipid content will lead to a reorganization of plasma membrane receptors and receptor‐associated scaffold resulting in signaling alterations of diverse pathways, including the insulin pathway. A number of observations support this prediction. First, several cell types of diabetic individuals present changes in the ratios of cholesterol, sphingomyelin, and saturated fatty acids (reviewed in Pilon, 2016). Second, the hippocampi of old rodents and humans present significant changes in lipid composition, particularly a reduction in cholesterol (Colin et al., 2016; Haughey, Bandaru, Bae, & Mattson, 2010; Svennerholm, Boström, Jungbjer, & Olsson, 1994). Third, the decrease in neuronal cholesterol has also been observed in murine models of T2D (Suzuki et al., 2010; Suzuki, Ferris, Chee, Maratos‐Flier, & Kahn, 2013). In support of a cause–effect relationship, two recent studies have shown that the mild reduction of cholesterol in the hippocampus is involved in the cognitive deficits of old mice, via inhibiting AMPA receptor internalization and reducing the transcription of learning and memory genes in response to a cognitive stimulus (Martin et al., 2014); Palomer et al., 2016). Moreover, in models of T2D, the reduction of neuronal cholesterol plays a decisive role in the loss of cognitive capacity in this situation (Suzuki, Ferris, Chee, Maratos‐Flier, & Kahn, 2013).

## 2 RESULTS

**Figure 1:**
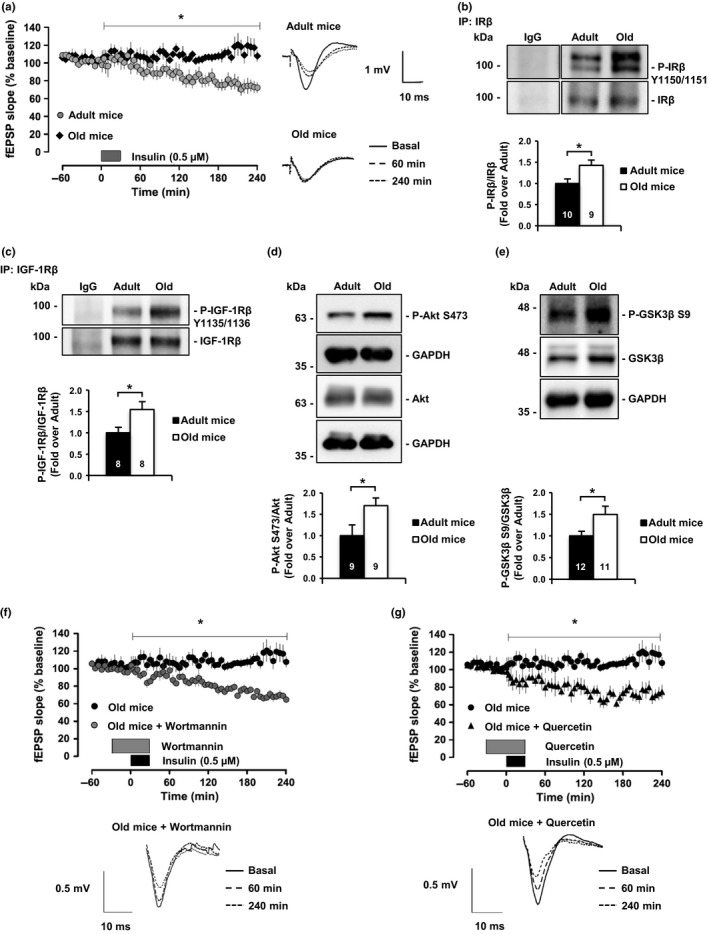
Insulin signaling is impaired in old mice due to PI3K/Akt hyperactivation. (a) Insulin‐LTD is completely abolished in old mice (20–24 months old; *n* = 7) compared to adult mice (7–12 months old; *n* = 10). The graphic represents insulin‐LTD as fEPSP slope. Gray box indicates the time of insulin application. Representative analogue traces on the right were collected at the indicated time points. (b) Insulin receptor (IR) basal activity levels detected by Western blot using Phospho‐Tyrosine 1150/1151 antibody after total protein immunoprecipitation. (c) Western blots reflect IGF‐1R Phospho‐Tyrosine 1135/1136 basal levels after total protein immunoprecipitation. Old mice hippocampus shows higher basal activity levels of IR and IGF1‐R compared to adult mice. (d) Akt Phospho‐Serine 473 activating residue detected by Western blot in adult and old mice hippocampus at basal level. (e) Western blot analysis of GSK3β in hippocampal extracts from adult and old mice. Consistent with Akt hyperactivation, the old hippocampus presents high GSK3β inhibitory mark (Phospho‐Serine 9). (f, g) PI3K/Akt inhibition rescues insulin‐LTD in old mice. Graphics showing insulin‐LTD induction in hippocampal slices from old mice incubated with Wortmannin (f; 0.5 μM; *n* = 7) and Quercetin (g; 20 μM; *n* = 8). Black box indicates time of insulin application. Gray box indicates time of inhibitors application. Representative analogue traces below were collected at the indicated time points. Numbers in bars reflect number of independent experiments. Data are represented as mean ± *SEM*. *t* test for (b, c, d, e), one‐way ANOVA with post hoc Bonferroni's test for (a, f, g). The asterisks *p* values (*p < 0.05; **p < 0.01; ns = not significant)

Numerous studies have demonstrated the existence of a deficit in insulin function in aged brains. However, it is not known how these defects affect insulin‐dependent synaptic functions. We observed that insulin‐induced long‐term depression (insulin‐LTD) was impaired in old mice (20–24 months old) compared to adult mice (7–12 months old; Figure 1a). To gain insight into how this insulin deficit occurs, we performed an immunoprecipitation‐based analysis of receptor activity in the hippocampus of adult and old mice in basal conditions. This experiment revealed that both insulin receptor (IR) and insulin‐like growth factor 1 receptor (IGF‐1R) are constitutively expressed at a higher level in old mice compared to young ones (Figure 1b,c). Among other possible explanations, the deficit in insulin‐LTD (Figure 1a) in conditions of high receptor activity (Figure 1b,c) may be due to the high PI3K activity. This possibility finds support in the fact that activation of IR/IGF‐1R would have produced activation of PI3K, which would phosphorylate PIP2 to PIP3, in turn activating Akt to phosphorylate GSK3β on Serine residue 9 (an inhibitory effect), resulting in the inhibition of hippocampal LTD (Peineau et al., 2007). Consistent with this view, we observed higher levels of active Akt in the hippocampus of old mice compared to adult mice (Figure 1d), and also higher levels of Serine 9‐phosphorylated GSK3β (Figure 1e). Moreover, insulin was able to evoke LTD in hippocampal slices from old mice that were pre‐incubated with PI3K/Akt inhibitors, but not in vehicle‐treated control slices (Figure 1f,g).

Reduced insulin function in a cellular background of high levels of active receptor and post‐receptor (PI3K/Akt) phosphorylation speaks in favor of the hippocampus of old mice being in a state of insulin desensitization (Zick, 2001). In agreement, hippocampal activity levels of the Akt downstream targets mTOR, p70S6K, and the resulting negative feedback on PI3K‐adaptor protein IRS‐1 (inhibitory phosphorylation at Serine residues 632 and 307 and 1101; Zick, 2001) are higher in the hippocampus of old vs. adult mice (Supporting Information Figures S1A‐C and S7A,B). In addition, IRS‐2, another IRS family protein that has been previously related to disrupted plasticity in the hippocampus (Costello et al., 2012), shows a trend to be reduced in old mice compared to adult mice (Supporting Information Figure S7C). Finally, another result that supports the existence of a desensitized route during old age is the reduced activation of the insulin receptor in response to peripheral insulin stimulation (Supporting Information Figure S1D) (Fernandez & Torres‐Alemán, 2012). In summary, these results reveal that one reason for the decrease in insulin function in the aged brain is an increase in PI3K/Akt activity, in part due to the chronic activity of tyrosine kinase receptors, such as those for insulin and IGF‐1 (also shown below).

Numerous studies have described changes in brain cholesterol and their correlation with cognitive deficits during aging, and in brain disorders such as Alzheimer's disease and T2D (Kadish et al., 2009; Suzuki, Ferris, Chee, Maratos‐Flier, & Kahn, 2013; Suzuki et al., 2010). In previous works, we showed that in the mouse there is also a correlation between cognitive deficit and reduction of membrane cholesterol in the hippocampus (Martín et al., 2014; Palomer et al., 2016; Trovò, Van Veldhoven, Martín, & Dotti, 2011). These observations moved us to test whether hippocampal membrane cholesterol reduction could be involved in the insulin‐desensitized state. To test this possibility, we reduced cholesterol levels in cultured hippocampal neurons by cholesterol oxidase (Choox) treatment. Choox was used at a concentration leading to a mild (~20%) reduction of plasma membrane cholesterol, without affecting cell viability (our previous works: Brachet et al., 2015; Palomer et al., 2016). Choox‐mediated cholesterol reduction led to an increase in phosphorylated IR and IGF‐1R (Supporting Information Figure S2A). Furthermore, cholesterol reduction also resulted in higher phosphorylation levels of Akt (Supporting Information Figure S2B), mTOR (Supporting Information Figure S7D), p70S6K (Supporting Information Figure S2C), and IRS‐1 at Serine 632, 307, and 1,101 (Supporting Information Figures S2D,E, and S7E). Cholesterol reduction was also sufficient to lead to a reduction of IRS‐2 levels (Supporting Information Figure S7F). To assess whether downstream receptor activation by cholesterol reduction was due to activation of IRs, Choox‐treated neurons were incubated with OSI‐906, a competitive inhibitor of IR and IGF‐1R phosphorylation (Mulvihill et al., 2009). OSI‐906 treatment significantly reduced the cholesterol loss‐mediated activation of Akt and IRS‐1 at Serine 632 (Supporting Information Figure S3A,B), but did not completely abrogate it because reduced cholesterol also increases the activity of other tyrosine kinase receptors (RTKs) (Martin et al., 2008). Nonetheless, the loss of neuronal cholesterol in our model appears to be sufficiently powerful to induce desensitization of the insulin pathway.

**Figure 2:**
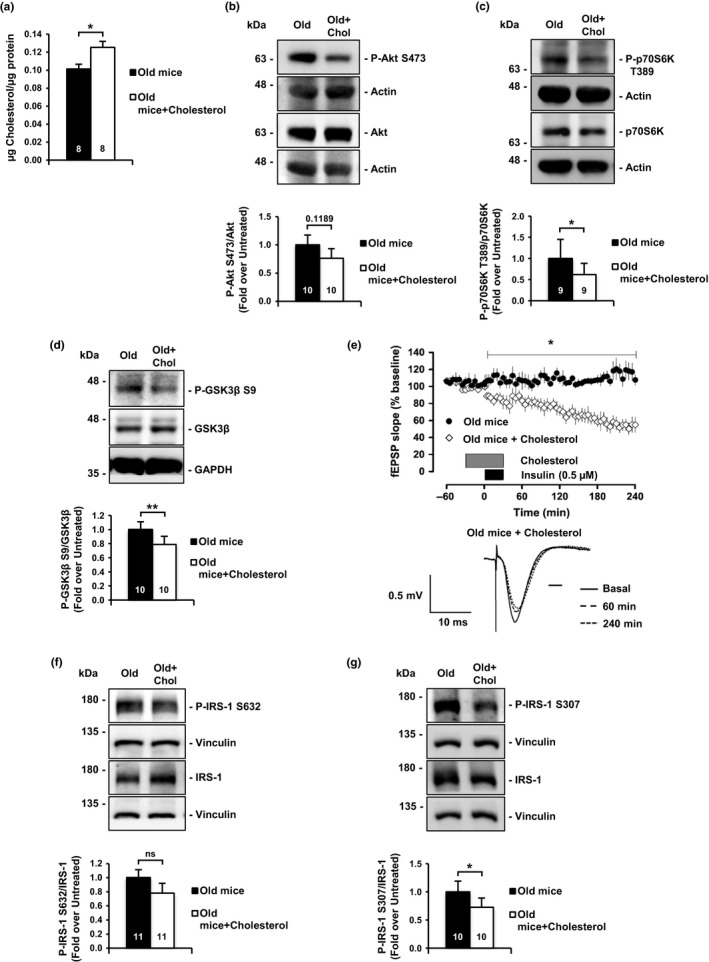
Ex vivo cholesterol replenishment in hippocampal slices of old mice restores insulin signaling. (a) Addition of cyclodextrin‐cholesterol (referred as MβCD‐Ch or Cholesterol) solution to old mice hippocampal slices induces a 20% increase in membrane cholesterol. (b, c) Cholesterol addition (as in a) reduces hyperactivated Akt and its downstream target p70S6K. (b) Detection of Akt Phospho‐Serine 473; (c) detection of p70S6K Phospho‐Threonine 389. (d) Cholesterol addition to old hippocampal slices reduces Phospho‐Serine 9 GSK3β inhibitory residue. (e) Addition of the cholesterol solution to hippocampal slices of old mice rescues the impairment of insulin‐LTD. Graphic showing insulin‐LTD induction in old mice slices (*n* = 7) and old mice slices incubated with MβCD‐Ch (*n* = 9). Black box indicates time of insulin application. Gray box indicates the time of MβCD‐Ch mix application. Representative analogue traces below were collected at the indicated time points. (f, g) Cholesterol replenishment decreases insulin resistance marks on IRS‐1 protein. (f) IRS‐1, Phospho‐Serine 632; (g) Phospho‐Serine 307. The value inside the bars indicates the number of independent experiments. Data are represented as mean ± *SEM*. *t* test for (a, b, d, g), Wilcoxon test for (c, f), one‐way ANOVA with post hoc Bonferroni's test for (e). The asterisks indicate the *p* values (*p < 0.05; **p < 0.01)

Next, we performed the converse experiments, adding cholesterol to old neurons. It has been previously shown that the high affinity of methyl‐beta‐cyclodextrin (MβCD) for cholesterol can be used to generate inclusion complexes (MβCD‐Ch) that increase membrane cholesterol levels (Zidovetzki & Levitan, 2007). Thus, hippocampal slices from old mice were incubated with MβCD‐Ch, following protocols used in a previous study in which we evaluated the role of membrane cholesterol in the internalization of glutamate receptors (Martin et al., 2014). The treatment restored cholesterol content to levels similar to those of adult mice (Figure 2a, see also ref. Palomer et al., 2016, and Supporting Information Figure S4A). Consistent with a causal relationship, cholesterol increase in the old slices reduced PI3K/Akt (Figure 2b), p70S6K Threonine residue 389 (Figure 2c), and GSK3β Serine 9 phosphorylation (Figure 2d), and rescued insulin‐LTD (Figure 2e). Furthermore, marks of insulin‐desensitized state on IRS‐1 (Serine 632 and Serine 307) were decreased in the extracts of the cholesterol‐treated slices (Figure 2f,g).

**Figure 3:**
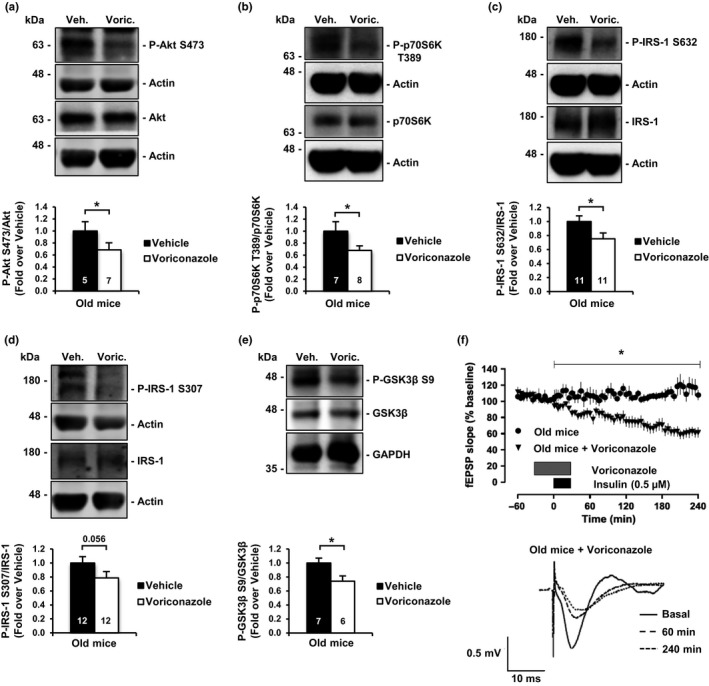
In vivo pharmacological inhibition of cholesterol loss in old mice restores hippocampal insulin sensitivity and insulin‐LTD. (a, b) Oral administration of the Cyp46A1 inhibitor Voriconazole reduces hippocampal PI3K/Akt and downstream effector p70S6K hyperactivation in old mice. (a) Western blot of Phospho‐Serine 473 activating residue on Akt. (b) Western blot of p70S6K Phospho‐Threonine 389. (c, d) Oral treatment with Voriconazole decreases insulin resistance marks in hippocampal samples of old mice. (c) Western blots for IRS‐1 resistance marks Phospho‐Serine 632; (d) Phospho‐Serine 307. (e) Voriconazole treatment reduces the inhibitory phosphorylation on GSK3β. (f) Graphic showing insulin‐LTD induction in old mice slices (*n* = 7) and old mice slices incubated with Voriconazole (*n* = 8). Hippocampal slices were incubated with Voriconazole 10 nM for 60 min starting 30 min before insulin stimulus. Black box indicates time of insulin application. Gray box indicates time of Voriconazole application. Representative analogue traces below were collected at the indicated time points. Bar graphs: Numbers inside indicate the number of independent experiments. Data are represented as mean ± SEM. *t* test for (a, b, c, d, e). One‐way ANOVA with post hoc Bonferroni's test for (*F*). The asterisks indicate the *p* values (*p < 0.05)

In order to give in vivo relevance to the above results, we interfered with age‐associated cholesterol loss by inhibiting Cyp46A1, the main catabolic enzyme for cholesterol in the brain (Russell, Halford, Ramirez, Shah, & Kotti, 2009). To that end, the effects of Voriconazole, a specific Cyp46A1 inhibitor capable of crossing the blood–brain barrier, were determined (Shafaati et al., 2010). The oral administration of Voriconazole for 40 days to 20‐month‐old mice significantly reduced age‐associated cholesterol loss compared to untreated old mice (Supporting Information Figure S4A,B), confirming our previous results (Palomer et al., 2016). Biochemically, hippocampal fractions from the treated mice showed significantly reduced levels of PI3K/Akt and the downstream effector p70S6K compared to untreated old mice (Figure 3a,b). Voriconazole treatment also resulted in a reduction of insulin resistance marks on IRS‐1 (Figure 3c,d) and the LTD‐inhibitory GSK3β Serine 9 phosphorylation (Figure 3e). At the electrophysiological level, application of Voriconazole to hippocampal slices of old mice restored insulin‐LTD (Figure 3f). All in all, these results are consistent with the possibility that the decrease in brain cholesterol that occurs with age plays an important role in the impaired brain insulin function of the old. As cells of other organs and tissues undergo changes in lipid composition during aging, and even more so in the case of T2D (Bakan et al., 2006; Pilon, 2016), insulin resistance of peripheral organs might be caused, at least in part, by similar mechanism as those in the hippocampus of old mice.

One possible explanation for how a reduced cholesterol environment could lead to steady receptor activity is by a tighter packaging of the receptor in liquid‐ordered membrane microdomains (i.e., rafts). Consistent with this possibility, we observed a significant increase of IR in the detergent‐insoluble fraction of hippocampal membranes from old mice compared to adult mice (Supporting Information Figure S5A). Furthermore, in a preliminary experiment we observed reduced levels of activity of Akt in the raft fraction of old mice that had been treated during 40 days with the Cyp46A1 inhibitor Voriconazole (Supporting Information Figure S5B), and therefore with levels of cholesterol similar to younger mice (see Supporting Information Figure S4A,B). Together, these last series of results suggest that aging increases IR clustering and activity in the raft domain of hippocampal cells. The obvious question that follows is: how is it possible that there is more receptor activity in the raft fraction in a membrane with reduced cholesterol?

In principle, the age‐dependent loss of cholesterol, a membrane rigidity molecule, is inconsistent with the possibility of increased IR partitioning in ordered domains such as rafts. However, this could be explained from the increase in plasma membrane sphingomyelin (SM), also a determinant of plasma membrane rigidity (Colin et al., 2016), whose levels increase with age and as a consequence of cholesterol reduction (Colin et al., 2016; Trovò, Van Veldhoven, Martín, & Dotti, 2011). In support of this possibility, previous works demonstrated an increase in TrkB receptor clustering in rafts in the plasma membrane of old neurons in a cholesterol‐reduced (Martin et al., 2008) and SM‐increased (Trovò, Van Veldhoven, Martín, & Dotti, 2011) manner. Moreover, also AMPA receptors are trapped in the membrane raft domain of old neurons due to reduced cholesterol (Martin et al., 2014). Thus, it appears that aging disturbs the plasma membrane so that new lipid rafts appear. This phenomenon can also be seen in cultured neurons of different ages (14 and 28 days) stained with di‐4‐ANEPPDHQ, a ratiometric dye that allows quantification of liquid order (Supporting Information Figure S5C; Amaro, Reina, Hof, Eggeling, & Sezgin, 2017; Owen, Rentero, Magenau, Abu‐Siniyeh, & Gaus, 2012). Although this result is from an “aging‐in‐the‐dish” experiment, it is noteworthy that in vitro “aged” hippocampal neurons present numerous changes that are typical of old hippocampal neurons in vivo, including but not limited to the gradual accumulation of senescence markers and a steady, significant reduction of cholesterol due to the activation of the Cyp46A1 enzyme (Martin et al., 2008; Sodero et al., 2011).

**Figure 4:**
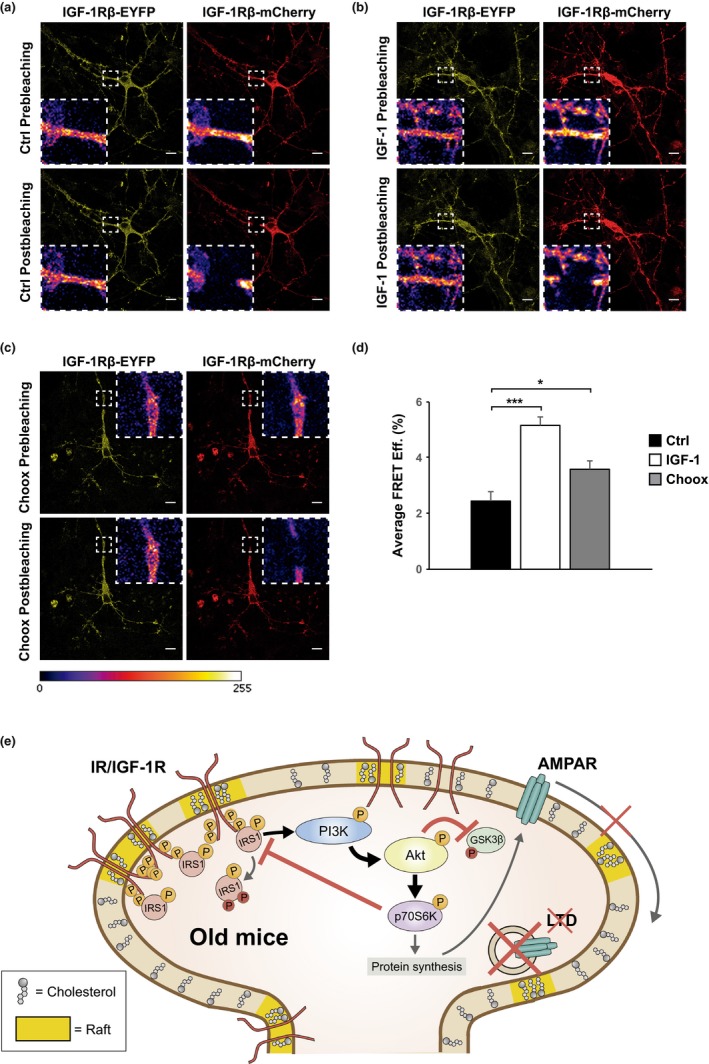
Cholesterol loss favors in neurons the conformational changes required for insulin growth factor 1 receptor's activation. (a, b, c) FRET experiment. Laser microscopy images of cultured neurons co‐transfected with IGF‐1R‐EYFP (donor fluorophore) and IGF‐1R‐mCherry (acceptor fluorophore), with the fluorophores replacing the cytosolic tails of the entire receptor sequence. Neurons maintained in Neurobasal+B27 (without serum) remained un‐stimulated (a), were stimulated with 4 μM IGF‐1 (b), or were treated with 10 IU/ml Choox before fixation (c). Acceptor signal was bleached, intensity of donor signal was measured, and FRET efficiencies were calculated (see Materials and Methods). Scale bars represent 10 μm. (d) Cholesterol loss enables in neurons a plasma membrane environment sufficient to induce the conformational change on IGF‐1R required for receptor activation, thus increasing FRET efficiency. The graphic represents FRET efficiencies data obtained from 80 images quantified of four independent experiments for each condition. Data are represented as mean ± *SEM*. One‐way ANOVA with post hoc Bonferroni's test for (a, b, c). The asterisks indicate the *p* values (*p < 0.05; ***p < 0.001). (e) Summary of how age produces insulin resistance in the mouse hippocampus: cholesterol loss, due in part to Cyp46a1 activation (and also to SCAP reduction, and perhaps others), favors an increase in plasma membrane ordered domains where the IR/IGF‐1R preferentially cluster (membrane yellow segments). This clustering facilitates the conformational changes required for the receptor's autophosphorylation in a ligand‐independent manner (phosphor motifs in IR/IGF‐1R). If this type of receptor change occurs over a prolonged period of time and incrementally (as it is the case with the loss of cholesterol with age), the post‐receptor pathway will also be activated chronically, leading to desensitization (phospho‐motifs in IRS‐1) and loss of insulin synaptic plasticity function (Akt inhibition on GSK3β and AMPAR inhibited internalization)

The above results led us to speculate that the cholesterol loss observed in old neurons could facilitate insulin/IGF‐1 activation by physically packaging the receptor subunit in the ordered domains, enabling thereby ligand‐independent autophosphorylation. In order to test this possibility, we transfected cells with full‐length IGF‐1R constructs bearing fluorescent donor or acceptor tags in the intracellular domain (as in Kavran et al., 2014), followed by fluorescence resonance energy transfer (FRET) analysis. As shown in the previous sections, both IR and IGF‐1R present the same age‐associated activity increase in a cholesterol‐dependent manner, suggesting that the FRET results based on IGF‐1R should be comparable to what occurs with IR and perhaps any other RTK. Previous work has shown that both receptors require insulin or IGF‐1 binding to their extracellular domain in order to become active, inducing a conformational change in the transmembrane domain leading to physical approach and autophosphorylation of the cytoplasmic tails (Kavran et al., 2014; Lemmon & Schlessinger, 2010). Previously established acceptor photobleaching FRET protocols allowed us to assess cytoplasmic tail proximity in response to different stimuli. Specifically, the donor intensity upon acceptor photobleaching and transfer of energy between fluorescent molecules is a measure of their proximity (see Section 4). To determine whether cholesterol loss could be considered a general mechanism of ligand‐independent receptor activation, we performed FRET experiments in two cell types, Hek‐293T and hippocampal neurons. In Hek‐293T cells, addition of the receptor–ligand (IGF‐1) caused the expected increase in FRET efficiency (Supporting Information Figure S6A,B). In order to test whether cholesterol loss similarly increased FRET, which would support induction by a similar conformational change, IGF‐1R‐transfected cells were treated with Choox as in the previous experiments (see Supporting Information Figure S2). Cholesterol reduction was sufficient per se to significantly increase the receptor's FRET efficiency (Supporting Information Figure S6C), though at a lower magnitude than addition of the natural ligand (see quantification in Supporting Information Figure S6D). This was expected, consistent with the slow development of insulin resistance parallel to the slow progression of brain cholesterol changes. Next, we repeated the experiment in cultured hippocampal neurons, transfected with the same IGF‐1R carrying EYFP and mCherry tags. Addition of IGF‐1 led to high FRET efficiency relative to untreated neurons (Figure 4a,b), with similar results in neurons treated with Choox in the absence of exogenous ligand (Figure 4c). However, as observed in Hek‐293T cells, the magnitude of the FRET increase was lower in conditions of low cholesterol compared to the ligand (Figure 4d).

## 3 DISCUSSION

Previous studies, in humans and in mice, point in the direction that some of the cognitive deficits of the old are due, among other causes, to the decreased levels or activity of insulin and IGF‐1 receptors and to a reduction in the signaling pathway by PI3K/Akt (Deak & Sonntag, 2012; Zaia & Piantanelli, 2000). This is quite different from the increased tonic receptor activity in the hippocampus of old mice, and increased PI3K/Akt pathway that we show here. The differences could be due to (perhaps too simplistic) the type of cellular or animal model, gender, or age utilized in the different works. On the other hand, given that in both studies the final result is loss of insulin signaling, it is not unrealistic to hypothesize that the different results simply reflect two stages of the same process: that is, increased basal receptor/PI3K‐Akt activity (as shown here) followed by reduction in the levels and sensitivity of Insulin/IGF‐1 receptors (as observed in previous works). More work is needed to determine whether this hypothesis is correct.

In addition to demonstrating that the insulin and IGF‐1 receptors are tonically active in the old, we also present evidence that this might be the consequence of the age‐associated alteration in the neuronal plasma membrane fluidity due to the reduction in plasma membrane cholesterol content. Although numerous studies have described changes in brain cholesterol and their correlation with cognitive deficits during aging, and in brain disorders such as Alzheimer's disease and T2D (Kadish et al., 2009; Martín et al., 2014; Palomer et al., 2016; Suzuki, Ferris, Chee, Maratos‐Flier, & Kahn, 2013; Suzuki et al., 2010), an association between changes in brain cholesterol and brain insulin resistance has not been established before. In this work, we present two series of results that support such a causal link: (a) The loss of insulin function in old mice can be rescued by either inhibition of cholesterol loss in vivo or by the addition of cholesterol to hippocampal slices from old mice, and (b) the removal of cholesterol in young neurons is sufficient to induce insulin resistance. At a finer mechanistic level, our data are consistent with the notion that a reduction in the cholesterol content in the plasma membrane produces molecular rearrangements that favor receptors' spontaneous (ligand‐independent) activity, for example, by increasing the proximity of the receptor transmembrane and cytoplasmic domains, inducing their autophosphorylation and activation (Figure 4e) in a manner similar to the addition of its natural ligand (Kavran et al., 2014; Lemmon & Schlessinger, 2010). This is supported, indirectly, by the higher number of active IR in the detergent‐insoluble domain of hippocampal membranes from old mice (see Supporting Information Figure S5A). The above conclusion also finds direct support from the increased fluorescence recovery of the donor–receptor after photobleaching of the acceptor in cells treated with Choox (see Figure 4). Despite the severe limitations of FRET experiments performed in transfected cells exposed to an acute cholesterol reduction strategy, they provide a possible explanation for how a lack of cholesterol could lead to chronic activation of IR/IGF‐1R receptors and pathway desensitization. This does not exclude that also other mechanisms such as hyperinsulinemia may contribute to such chronic receptor activity.

In addition to increased receptor and post‐receptor (PI3K/Akt) activity, the loss of cholesterol also produces an increase in activity of mTORC1. This last result contradicts recent studies that show that cholesterol increases mTORC1 activity (Castellano et al., 2017). As before, there are substantial differences in the experimental models that can easily explain the divergent results (i.e., cell lines vs. hippocampal neurons, in vitro and in vivo, lysosomal mTORC1 vs. cytosolic mTORC1). Irrespective, our demonstration of the existence of a strong PI3K/Akt activity in the old hippocampus and in conditions of cholesterol loss (of note: this was also reported in Martín et al., 2014 and in Trovò et al., 2013) makes us feel confident that aging increases mTORC1 activity in a cholesterol loss/Akt‐dependent manner and that this can be responsible in part for the negative phosphorylation of IRS‐1 and desensitization of the insulin signaling pathway. In further agreement, the increase in mTORC1 activity in the old hippocampus and in cultured hippocampal neurons with reduced cholesterol could explain the age and cholesterol loss‐dependent increase in P‐p70S6K and in IRS‐1‐negative phosphorylation.

There may seem to be a contradiction between desensitization of the insulin pathway and high basal activity levels of the PI3K/Akt pathway, as the activity of the latter depends on the former. However, it is worth remembering that PI3K/Akt activity is a common effector of numerous intracellular pathways, many of which are potentiated during aging, including interleukin production and activation of other RTKs (Martin et al., 2008; Xiao, Peng, Gan, Arafat, & Yin, 2016). This “normal” increase of PI3K/Akt in the cytoplasm of old neurons would explain why suppression of PI3K activity rescues LTD upon addition of insulin. Given the fact that the PI3K/Akt cascade is one of the important cell survival pathways (Kim et al., 2002; Miyawaki et al., 2009), neuronal survival mechanisms seem to be strengthened during aging and this redefinition of metabolic/organismic priorities occurs to the detriment of other cellular functions, such as LTD. On the other hand, the high PI3K/Akt activity observed in the old hippocampus may be the reason why age does not largely affect the magnitude of LTP (reviewed in Kumar, 2011), a process highly dependent on PI3K/Akt. Only future work can bring more light to how the increased activity of the PI3K‐Akt pathway in the old brain participates in the balance between LTP and LTD in the hippocampus and between plasticity and survival. Thus, we believe that besides helping to understand how insulin function becomes reduced in the brain with age, our work also opens ways to investigate essential events of aging, such as the relationship between survival and plasticity.

## 4 EXPERIMENTAL PROCEDURES

### 4.1 Animal handling

Male adult (7–12 months old) and old (20–24 months old) C57BL/6J mice were used in this study. All the animals were kept in the Centro de Biología Molecular Severo Ochoa (CBMSO) animal facility. All experiments were performed in accordance with European Union guidelines (2010/63/UE) regarding the use of laboratory animals.

### 4.2 Animal treatment

Voriconazole (from Hangzhou Dayangchem Co., China CAS No: 137234‐62‐9) was solubilized using (2‐hydroxypropyl)‐β‐cyclodextrin (Sigma‐Aldrich, St Louis, MO): 15 g of (2‐hydroxypropyl)‐β‐cyclodextrin was dissolved in 100 ml of saline solution (0.9% NaCl) and heated to 80°C in a water bath with stirring. Then, 1.5 g of Voriconazole was added to the cyclodextrin solution with stirring at 80°C until complete dissolution. This stock solution (15 mg Voriconazole/ml cyclodextrin/saline) was conserved at 4°C protected from light. On the first day of experimentation, an aliquot of this solution was diluted in drinking water to a final concentration of 0.68 mg/ml Voriconazole. Considering that a mouse drinks 3 ml water per day, the dose corresponds to 2.04 mg/day. The average weight of a mouse is 34 g, resulting therefore in a dose 60 mg/kg body weight. Water with this concentration of Voriconazole was used as the hydration source in 20‐month‐old mice during 45 days. Vehicle 20‐month‐old mice received the same amount of (2‐hydroxypropyl)‐β‐cyclodextrin than Voriconazole mice during 45 days. Bottles containing the Voriconazole or vehicle water were changed weekly.

### 4.3 Hippocampal slices

Hippocampal slices were obtained from C57BL/6J mice. Hippocampi were extracted and placed in dissection solution (10 mM d‐glucose, 4 mM KCl, 26 mM NaHCO_3_, 233.7 mM sucrose, 5 mM MgCl_2_, 1:1,000 phenol red), oxygen saturated with carbogen (95% O2/5% CO_2_), and sliced using an automatic tissue chopper (McIlwain Tissue Chopper, Standard Table, 220 V; Ted Pella Inc., Redding, CA) to obtain 400 µm hippocampal slices. Then, slices were kept in artificial cerebrospinal fluid (ACSF: 119 mM NaCl, 2.5 mM KCl, 1 mM NaH_2_PO_4_, 11 mM glucose, 1.2 mM MgCl_2_, 2.5 mM CaCl_2_, osmolarity adjusted to 290 Osm), oxygen saturated with carbogen for 1 hr. Experiments were performed in ACSF, oxygen saturated with carbogen.

### 4.4 Cell cultures

Hek‐293T cells were grown in Dulbecco's modified Eagle's medium (DMEM) supplemented with 10% fetal bovine serum (FBS), 2 mM glutamine, 100 IU/ml penicillin, and 100 mg/ml streptomycin (complete DMEM). Primary hippocampal neurons were extracted from Wistar rat embryos at embryonic day 18 (E18), seeded in culture conditions as previously described (Kaech & Banker, 2006), and kept in culture for 15 days in vitro (DIV). All cells were incubated at 37ºC, humidity conditions, and 5% CO_2_.

### 4.5 Cell treatments

The following compounds were added to cell medium of hippocampal neurons and Hek‐293T: OSI‐906 (Selleckchem, Houston, TX ref.: S1091; 1 µM); Cholesterol oxidase (Choox; Calbiochem, San Diego, CA ref.: 228250; 10 IU/ml); human peptide purified IGF‐1 (Stem Cell Technologies, Canada #78022; 4 µM). For electrophysiology experiments we used: Insulin (Sigma‐Aldrich, St Louis, MO ref.: I0516; 0.5 µM); Quercetin (Sigma‐Aldrich, St Louis, MO ref.: Q4951; 20 µM); Wortmannin (Tocris, UK ref.: 1232; 0.5 µM); and Voriconazole (Hangzhou Dayangchem Co., China; 10 nM). Experiments for cholesterol addition conducted in hippocampal slices were performed at 25ºC. Methyl‐β‐cyclodextrin‐cholesterol (MβCD‐Ch) solution was prepared freshly at use concentration in ACSF, containing 30 µM Cholesterol Water‐soluble (Sigma‐Aldrich, St Louis, MO ref.: C4951) and 5 µM Cholesterol (Sigma‐Aldrich, St Louis, MO ref.: C3045).

### 4.6 Cholesterol quantification

Hippocampal extracts were homogenized in a lysis buffer containing 25 mM MES, 2 mM EDTA, Phosphatase Inhibitor Cocktail 2 (Sigma‐Aldrich), and Protease Inhibitor Complete (Roche, Switzerland). The protein amount was quantified by Pierce^®^ BCA Assay kit (Thermo Scientific, Waltham, MA) and the cholesterol content measured per microgram of protein using the Amplex^®^ Red Cholesterol Assay kit (Invitrogen, Carlsbad, CA).

### 4.7 Antibodies

The following antibodies were used for Western blot (WB) and immunoprecipitation (IP): anti‐α‐Tubulin (WB 1:10,000, Abcam, Cambridge, UK ref.: ab7291), anti‐β‐Actin (WB 1:20,000; Sigma‐Aldrich ref.: A5441), anti‐GAPDH (WB 1:20,000; Abcam, ref.: ab8245), anti‐Akt (WB 1:1,000; Cell Signaling, Danver, MA ref.: #9272), anti–Phospho‐Akt Serine 473 (WB 1:1,000, Cell Signaling ref.: #4060), anti‐GSK3 α/β (WB 1:1,000, Invitrogen, Carlsbad, CA ref.: 44–610), anti‐Phospho GSK3 α/β Serine 21/9 (WB 1:1,000; Cell Signaling ref.: #9331), anti‐IGF‐1 Receptor β (WB 1:1,000, IP 1:100; Cell Signaling ref.: #9750), anti‐Phospho IGF‐1 receptor β Tyr1135/116 (WB 1:1,000; Cell Signaling ref.: #3024), anti‐Insulin receptor β (IP 1:100, Santa Cruz, Dallas, TX ref.: sc‐57342), anti‐Insulin receptor β (WB 1:750, Santa Cruz, Dallas, TX ref.: sc‐711), anti‐Phospho insulin receptor β Tyr1150/1151 (WB 1:750; Millipore, Burlington, MA ref.: 04‐299), anti‐IRS‐1 (WB 1:1,000; BD Biosciences, Franklin Lakes, NJ ref.: 611394), anti‐Phospho IRS‐1 Serine 307 (WB 1:750; Abcam ref.: ab4865), anti‐Phospho IRS‐1 Serine 632 (WB 1:200; Cell Signaling ref.: #2388), anti‐Phospho IRS‐1 Serine 1101 (WB 1:1,000; Cell Signaling ref.: #2385), anti‐mTOR (WB 1:1,000; Cell Signaling ref.: #4517), anti‐Phospho mTOR Serine 2448 (WB 1:2,500; Cell Signaling ref.: #5536), anti‐p70S6K (WB 1:1,000; BD Biosciences ref.: 611260), anti‐Phospho p70S6K Threonine 389 (WB 1:750; Cell Signaling ref.: #9206), anti‐IRS‐2 (WB 1:500, Upstate 06‐506), anti‐Vinculin (WB 1:1,000; Millipore ref.: AB6039).

### 4.8 Insulin receptor sensitivity experiment

Insulin receptor sensitivity was determined in mouse hippocampus by measuring the capability of the receptor to become active upon ligand supply. Adult and old male mice were intraperitoneally injected with 27 IU/kg body weight of human purified insulin (Actrapid^®^, Novo Nordisk, Denmark) diluted in saline solution (0.9% NaCl). Injected mice were kept 1 hr at regular conditions and then sacrificed. Hippocampi were extracted and processed in RIPA buffer (20 mM Tris‐HCl, pH 7.5, 150 mM NaCl, 1 mM EDTA, 1 mM EGTA, 1% NP‐40, 1% sodium deoxycholate, 0.1% SDS, Phosphatase Inhibitor Cocktail 2 (Sigma‐Aldrich), and Protease Inhibitor Complete (Roche, Switzerland). The protein amount was quantified by Pierce^®^ BCA Assay kit. Samples were immunoprecipitated using an antibody against insulin receptor, and the level of receptor phosphorylation was determined by Western blot.

### 4.9 Insoluble vs. soluble membrane fraction isolation:

Extracts from adult and old mice hippocampi were incubated for 40 min at 4°C under rotation in TNE buffer (50 mM Tris–HCl, 150 mM NaCl, 5 mM EDTA [pH 7.4]) containing 1% Triton X‐100 and a protease inhibitor cocktail. The suspension was centrifuged at 184000 *g* rpm for 1 hr at 4°C. After centrifugation, the detergent‐insoluble membranes (raft) were collected from the pellet, whereas detergent soluble material (nonraft) was retrieved from the supernatant.

### 4.10 Raft fraction isolation

Mice hippocampal extracts were incubated at 4ºC for 45 min in 1% Triton X‐100, 10 mM MES (2‐[*N*‐morpholino]ethanesulfonic acid) (pH 7.00), 2 mM EDTA, 1 mM DTT, Phosphatase Inhibitor Cocktail 2 (Sigma‐Aldrich), and Protease Inhibitor Complete (Roche). Samples were brought to 60% sucrose. A discontinuous gradient was overlaid with 35% and 5% sucrose. After centrifugation at 100,000 *g* for 18 hr at 4ºC, 12 fractions were collected.

### 4.11 Fluorescence resonance energy transfer

Insulin‐like growth factor 1 receptor (IGF‐1R) activity was measured by fluorescence resonance energy transfer (FRET) in hippocampal neurons in culture or Hek‐293T transfected with IGF‐1R extracellular and transmembrane regions fused to EYFP (FRET donor) or mCherry (FRET acceptor). Plasmids were kindly provided by Dr. Patrick. O. Byrne and Dr. Daniel J. Leahy from Johns Hopkins University School of Medicine, Baltimore, USA. Neurons were transfected using Lipofectamine 2000 (Thermo Fisher ref.: #11668027). Hek‐293T cells were transfected using polyethylenimine (PEI) reagent (Polysciences, Warrington, PA ref.: #23966‐2). Forty‐eight hours later, cells were treated. Neurons were maintained in Neurobasal+B27 medium without serum for treatments. Hek‐293T cells required 5‐hr starvation in DMEM without FBS and glutamine previous to treatment. Different treatments were applied to determine the FRET efficiency: control situation (cells incubated only with starving medium), positive control situation (cells incubated with IGF‐1 peptide 4 µM), and study situation (cells incubated with Choox 10 IU/ml). After treatments, cells were fixed with 1% PFA for 15 min at room temperature. Finally, PFA was removed and cells were washed four times in 1× PBS and mounted onto slides using Mowiol–Dabco (Mowiol, Calbiochem, San Diego, CA) without antifading. A confocal LSM710 microscope (Zeiss, Germany) coupled to an inverted AxioObserver Z1 microscope (Zeiss) was used for conducting acceptor photobleaching FRET experiments. Images were acquired using the following wavelengths: *λ*_exc_^ ^= 488 nm, *λ*_em_^ ^= 503–532 nm for EYFP; *λ*_exc_ = 561 nm, *λ*_em_^ ^= 600–699 nm for mCherry. Three prebleaching and four postbleaching images were taken in both channels. Photobleaching was performed in delimited regions of interest (ROI) of the cells, in particular in the plasma membrane of the cells where the IGF‐1R is mainly located under physiological conditions. Images were taken using a 63× oil objective, and the ROIs used were squares of regular size. Acceptor signal was bleached up to a 10% of its initial magnitude applying the highest laser intensity during thirty iterations to the selected ROI. Zeiss imaging software was used to calculate FRET efficiency using the following equation:

$$\text{FRET}\;\text{Efficiency}\;\left(\%\right)\;=\;\left[\left({\text{Donor}}_{\text{post}}\;-\;{\text{Donor}}_{\text{pre}}\right)/{\text{Donor}}_{\text{post}}\right]\;\times \;100$$

where Donor_pre_ corresponds EYFP signal before bleaching, and Donor_post_ corresponds to final EYFP signal after bleaching. All images were background‐subtracted.

### 4.12 Electrophysiology (insulin‐LTD)

Adult and old mice were tested for synaptic plasticity in the hippocampal CA1 region in vitro. Animals were killed by cervical dislocation, and the hippocampus was rapidly dissected out into ice‐cold (4ºC) ACSF saturated with carbogen. ACSF for electrophysiology experiments consisted of: 124 mM NaCl, 4.9 mM KCl, 25.6 mM NaHCO_3_, 1.20 mM KH_2_PO_4_, 2 mM CaCl_2_, 2 mM MgSO_4_, 10 mM glucose, pH 7.4. Transverse slices (400 µm thick) were prepared from the dorsal area of the right hippocampus with a tissue chopper and placed into a submerged‐type chamber, where they were kept at 32ºC and continuously perfused with ACSF at a flow rate of 2.2 ml/min. After 90‐min incubation, one slice was arbitrarily selected and a tungsten electrode was placed in CA1 stratum radiatum. Field excitatory postsynaptic potentials (fEPSPs) were recorded by placing a glass electrode (filled with ACSF, 3–7 MΩ) in the stratum radiatum opposite the stimulating electrode. The time course of the field EPSP was measured as the descending slope function for all sets of experiments. After input/output curves had been established, the stimulation strength was adjusted to elicit a fEPSP slope of 35% of the maximum and kept constant throughout the experiment. During baseline recording, 3 single stimuli (0.1 ms pulse width; 10‐s interval) were measured every 5 min and averaged for the 60‐min fEPSP values. Unless otherwise stated, insulin‐mediated LTD was achieved by application of 0.5 µM insulin dissolved in ACSF for 30 min commencing at time zero (Huang et al., 2004). Drug or test substances were applied from 30 min prior to until 30 min after the start of insulin application. Evoked responses were initially monitored at 1, 4, 7, and 10 min and then continually every 5 min as above.

### 4.13 Plasma membrane order experiment

Rat hippocampal neurons were cultured 14 and 28 days in vitro (DIV) and fixed in 4% PFA. For lipid phase imaging, cells were stained with di‐4‐ANEPPDHQ (Thermo Fisher) according to the manufacturer's specifications. For imaging, we used a Nikon A1R confocal microscope attached to Ti eclipse outfitted with a Plan Apo VC 60× lens, oil immersion lens, with a NA of 1.4. For imaging, we used the spectral detector of the system set to 530–590 nm and 590–650 nm to image the spectral shift of the dye from lipid ordered to disordered phase. The resulting images were analyzed according to Owen Rentero Magenau Abu‐Siniyeh and Gaus (2012). To compare the two groups, the average value of 108 (60 for 14 DIV [12/coverslip, 5 experiments] and 48 for 28 DIV [12/coverslip, 4 experiments]) fields of view was reported.

### 4.14 Statistical analyses

Statistical analyses were performed with GraphPad Prism 5 (GraphPad Software Inc.). All values are presented as mean ± *SEM*. Data normality and variances were tested by the Shapiro–Wilk test. Mann–Whitney *U* test, Kruskal–Wallis test, or Friedman test, with Dunn's adjustment for multiple comparisons, was used for nonparametric data. Student's *t* test or ANOVA with Bonferroni's adjustment for multiple comparisons was used for parametric data. Asterisks in the figures indicate *p* values as follows: *<0.05; **<0.01; ***<0.001.

## CONFLICT OF INTEREST

None declared.

## AUTHOR CONTRIBUTIONS

A.M.‐S., T.A., D.B., and C.G.D. contributed to the design of the different experiments. A.M.‐S., T.A. A.K, S.M., E.P., I.P.‐P., and A.C.‐P. performed the experimental work. A.M.‐S., T.A., and D.B. did the statistical analysis. C.G.D. and D.B. prepared the manuscript with the help of all authors. C.G.D. is the guarantor of this study.
