# Supplementary figures and images for: Age‐associated cholesterol reduction triggers brain insulin resistance by facilitating ligand‐independent receptor activation and pathway desensitization
Source: Aging Cell. 2019 Mar 18;18(3):e12932. doi: 10.1111/acel.12932 (PMC6516156; doi:10.1111/acel.12932)

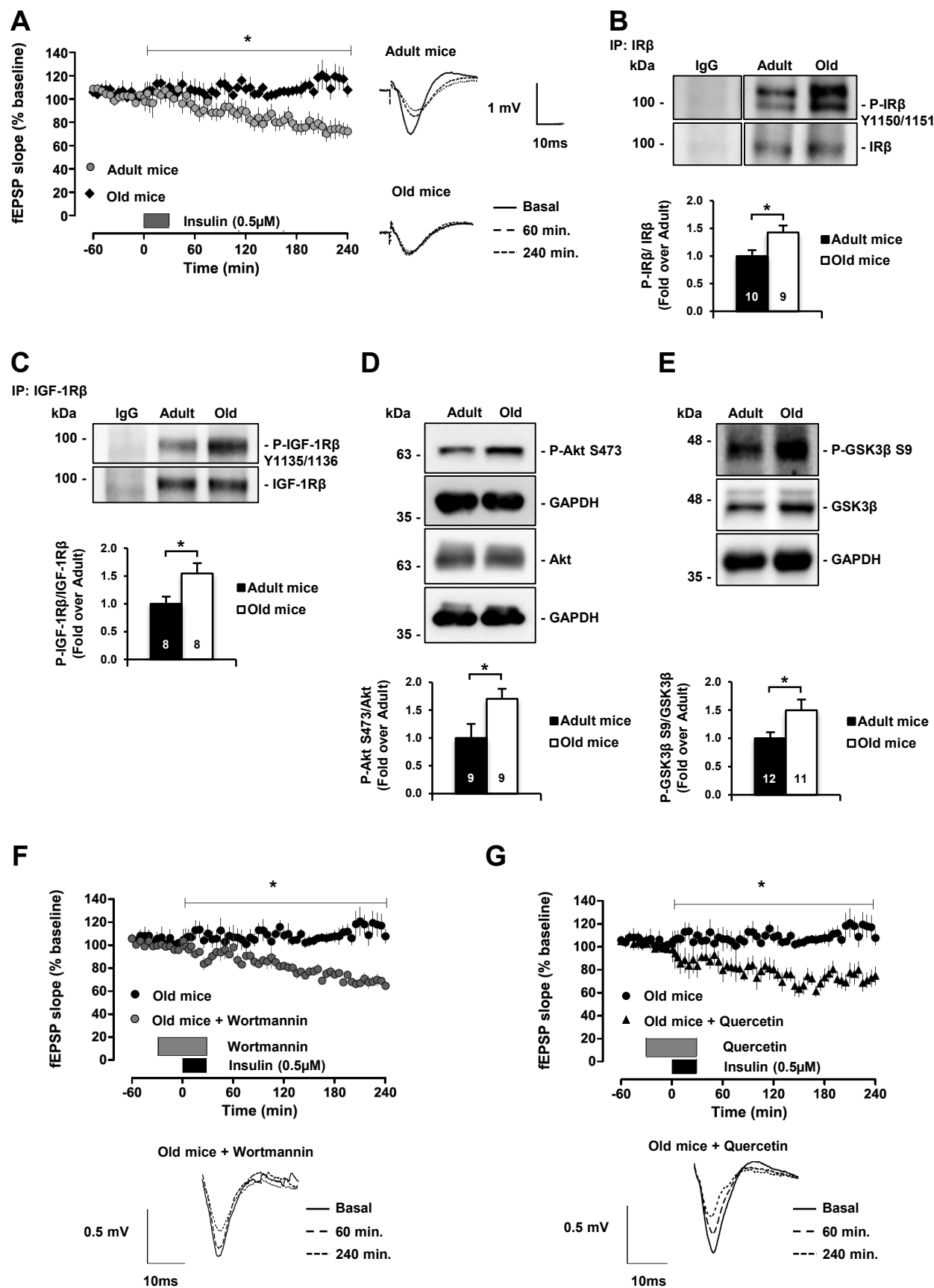

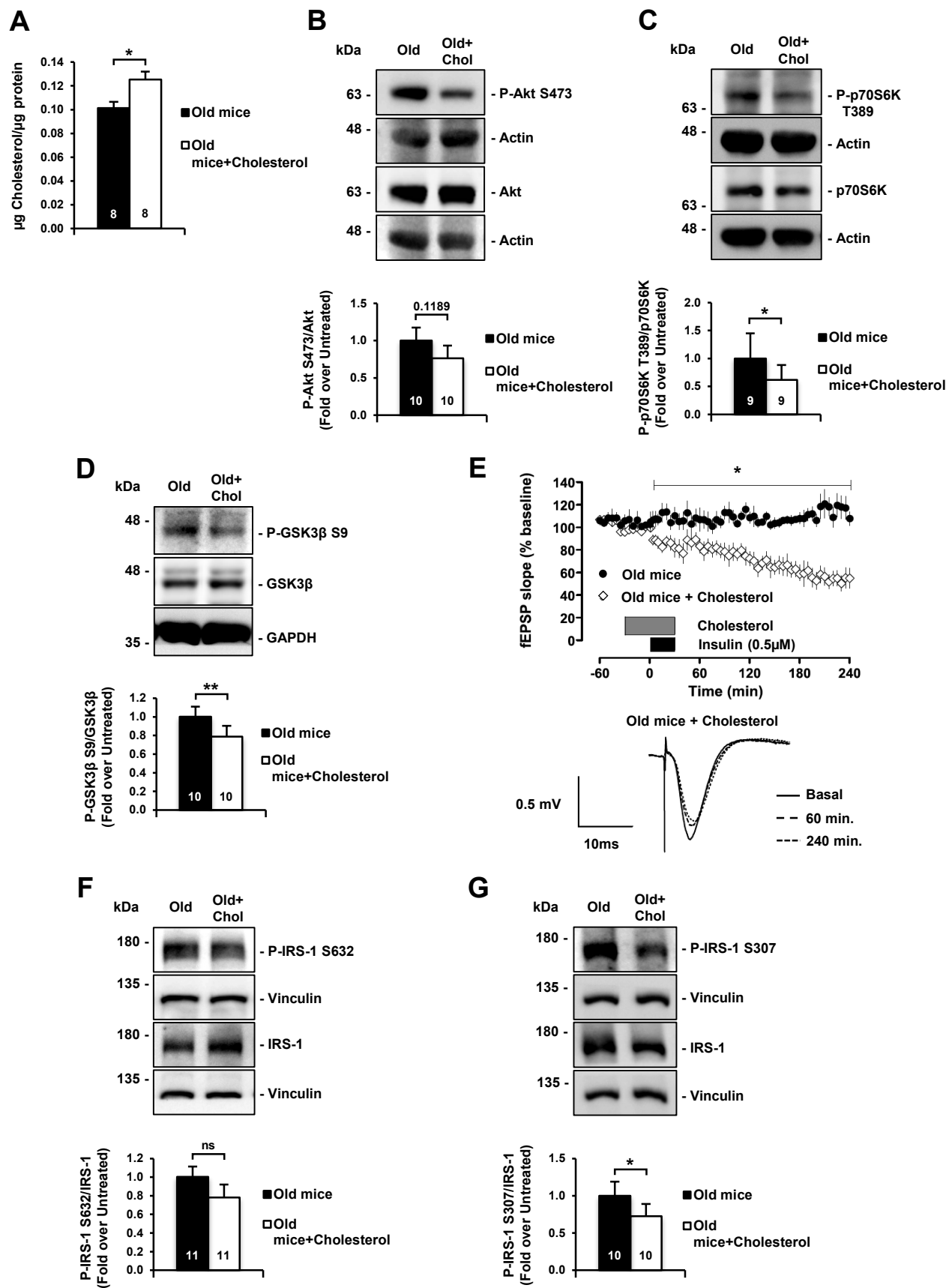

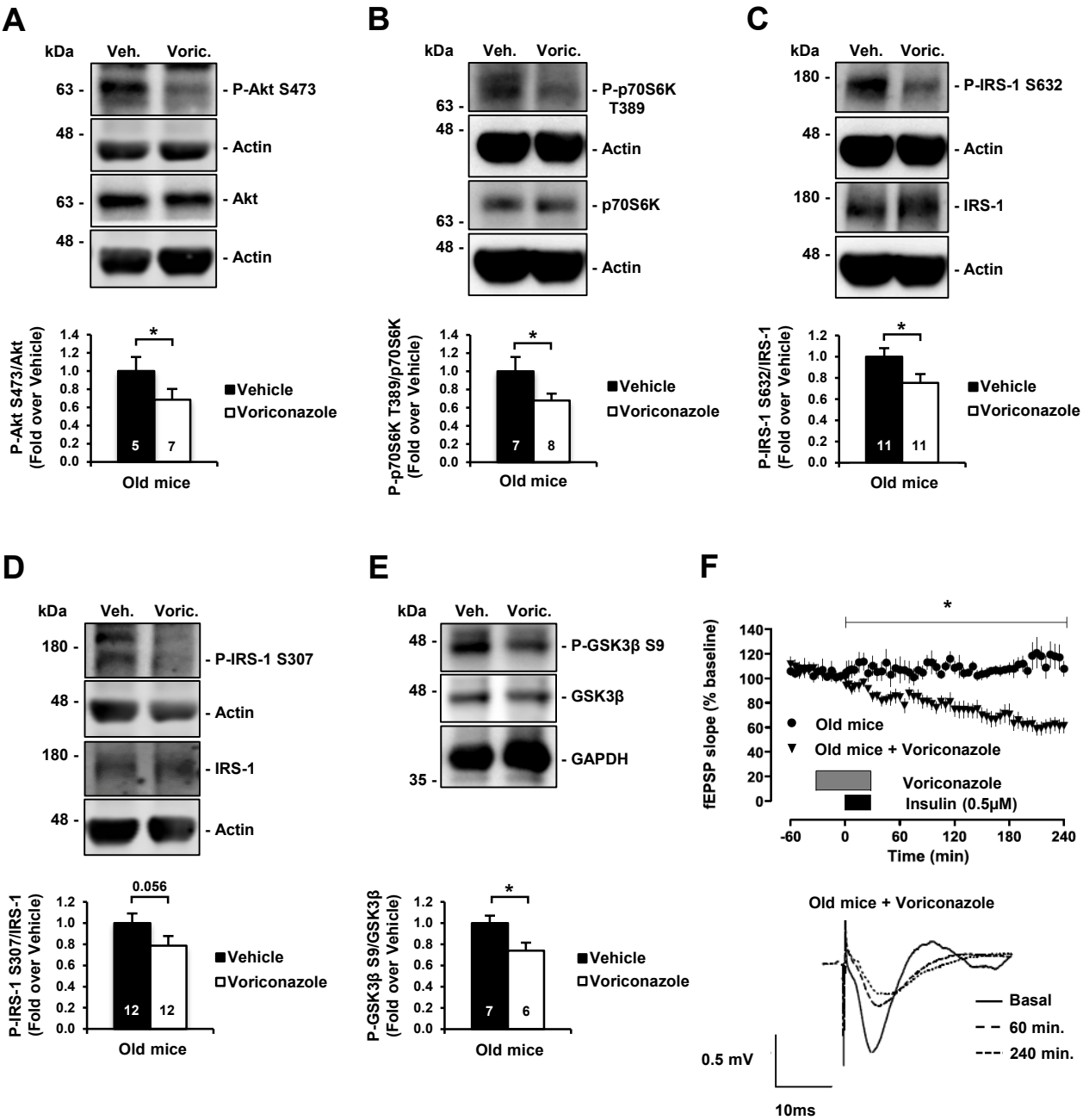

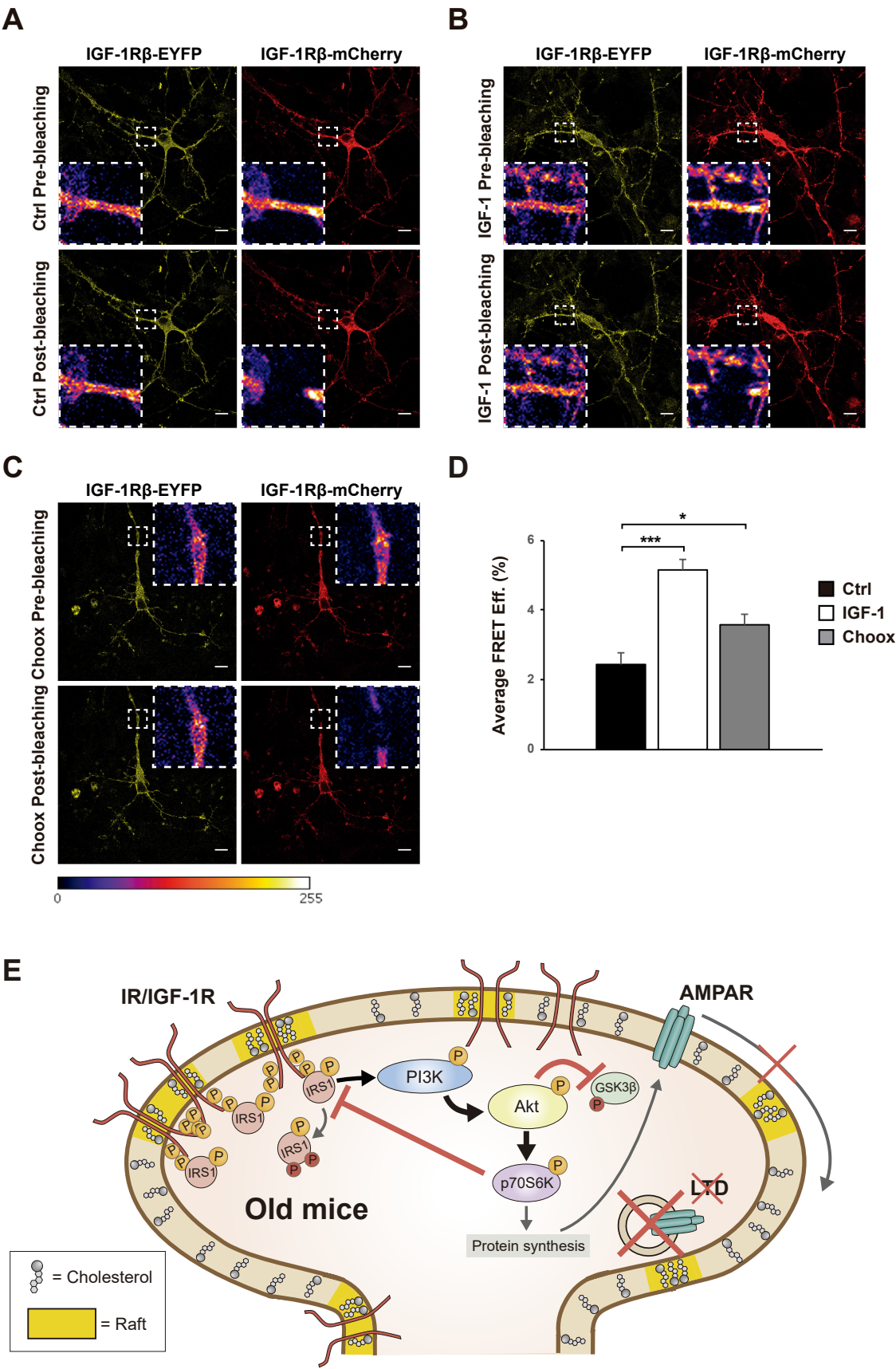

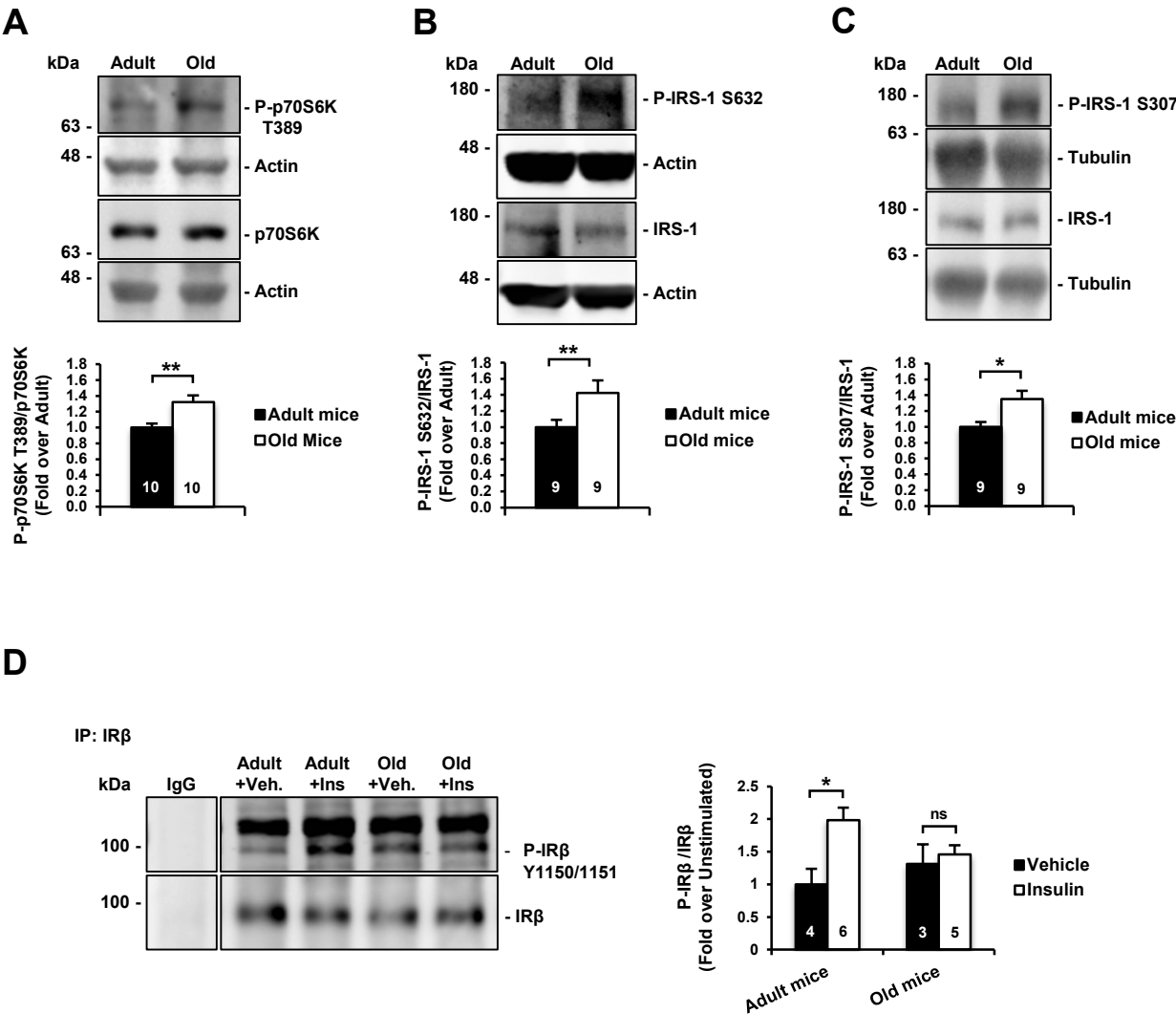

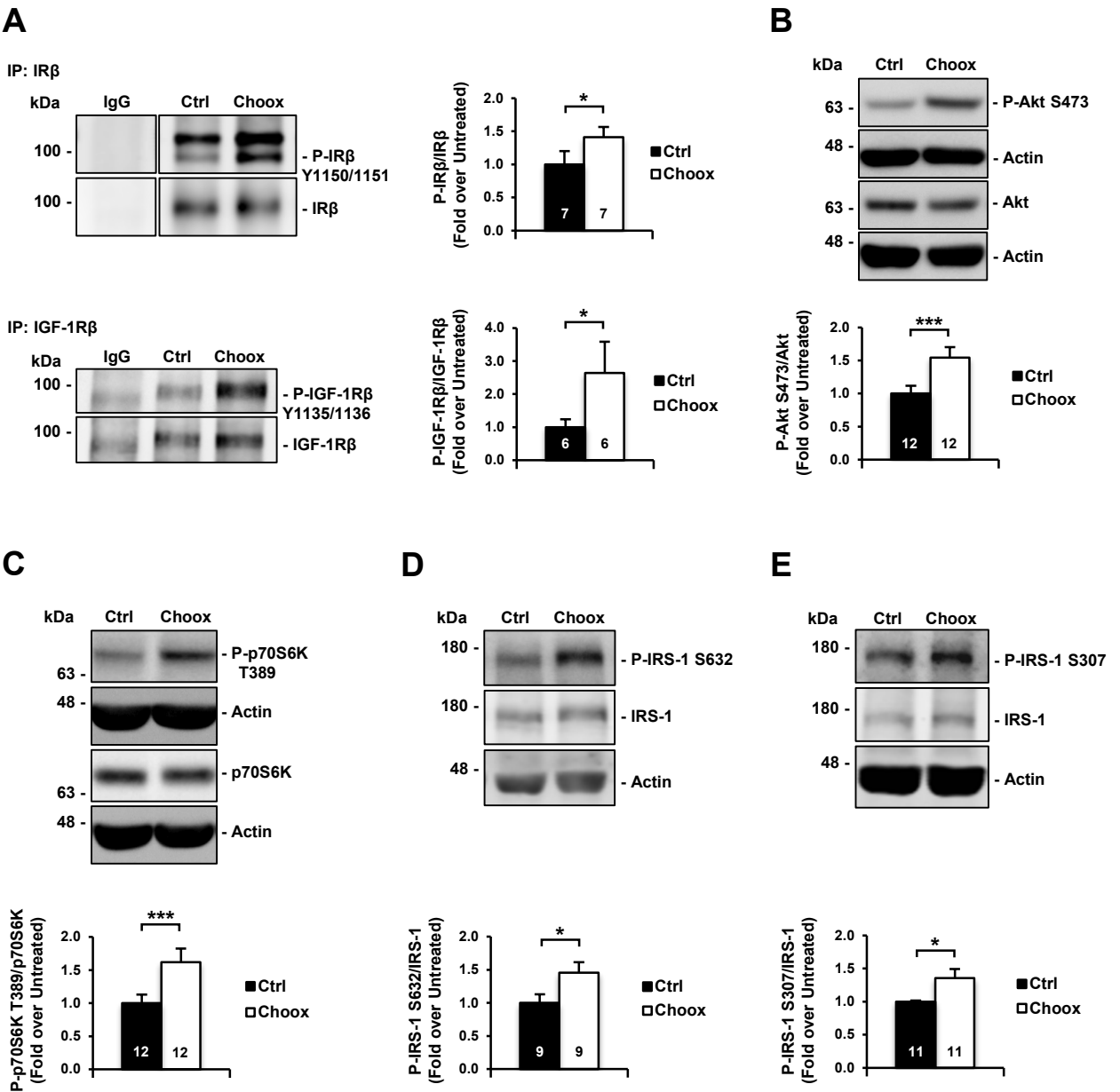

A

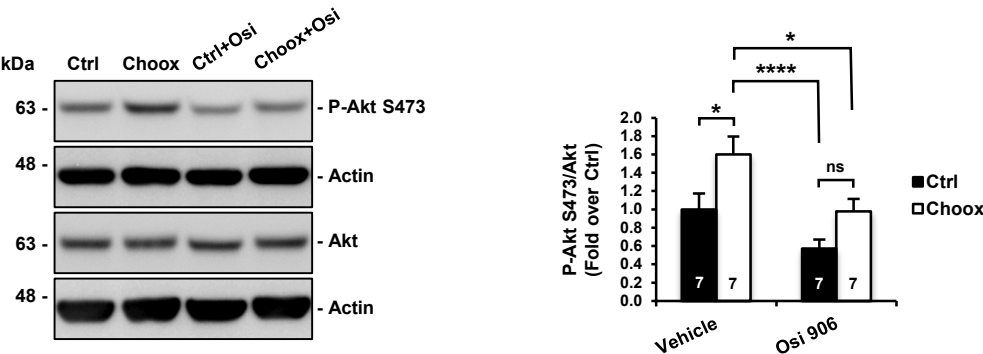

B

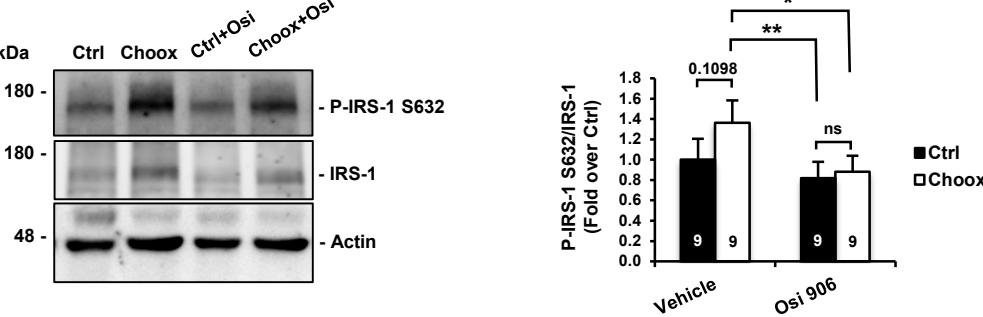

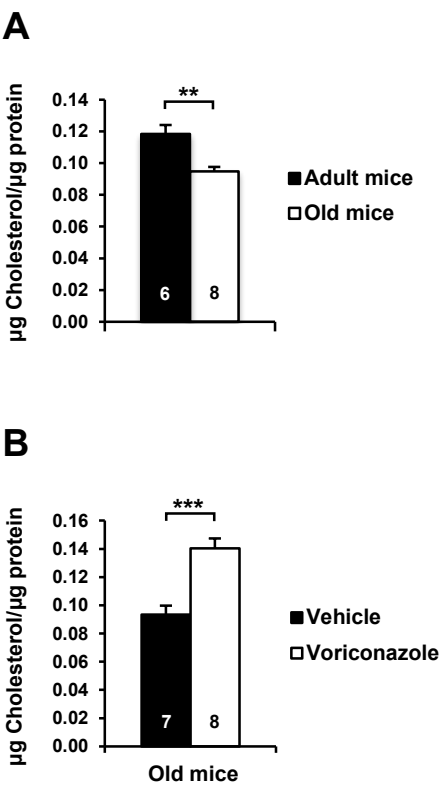

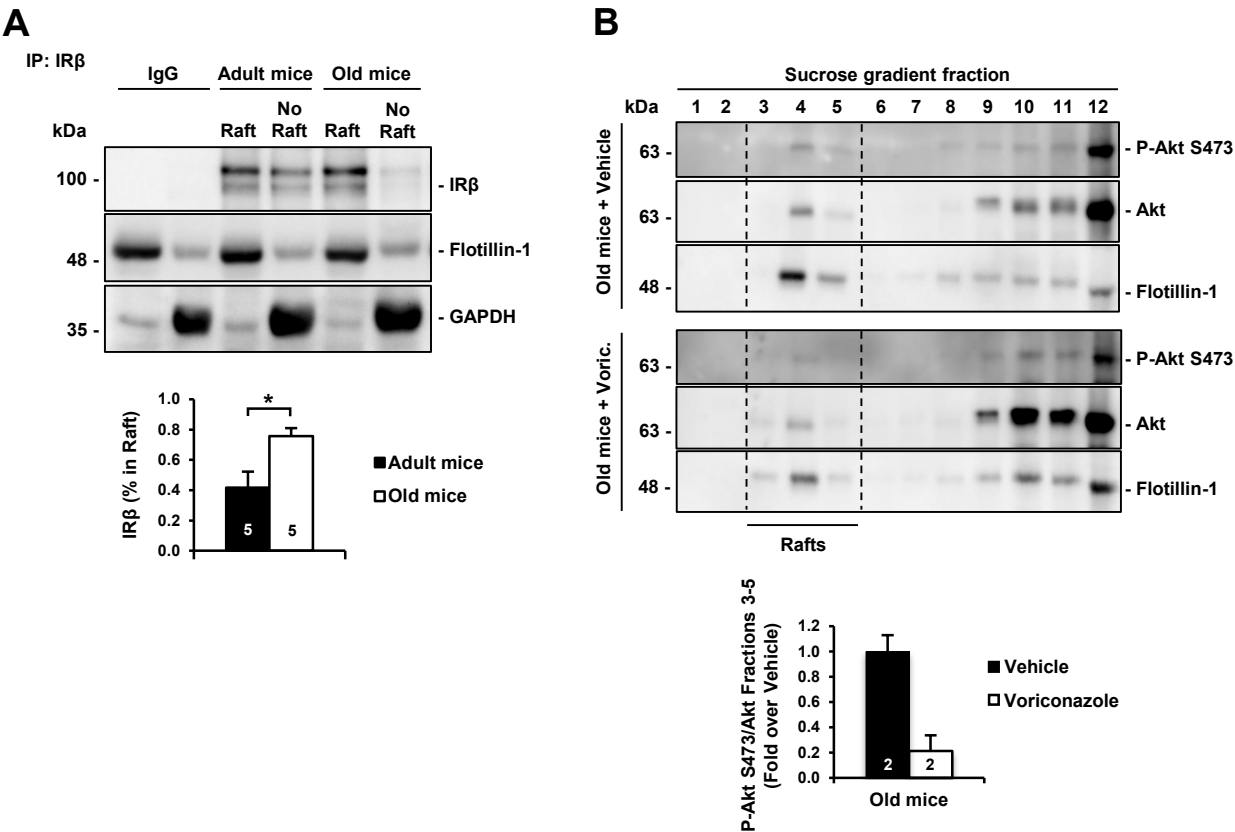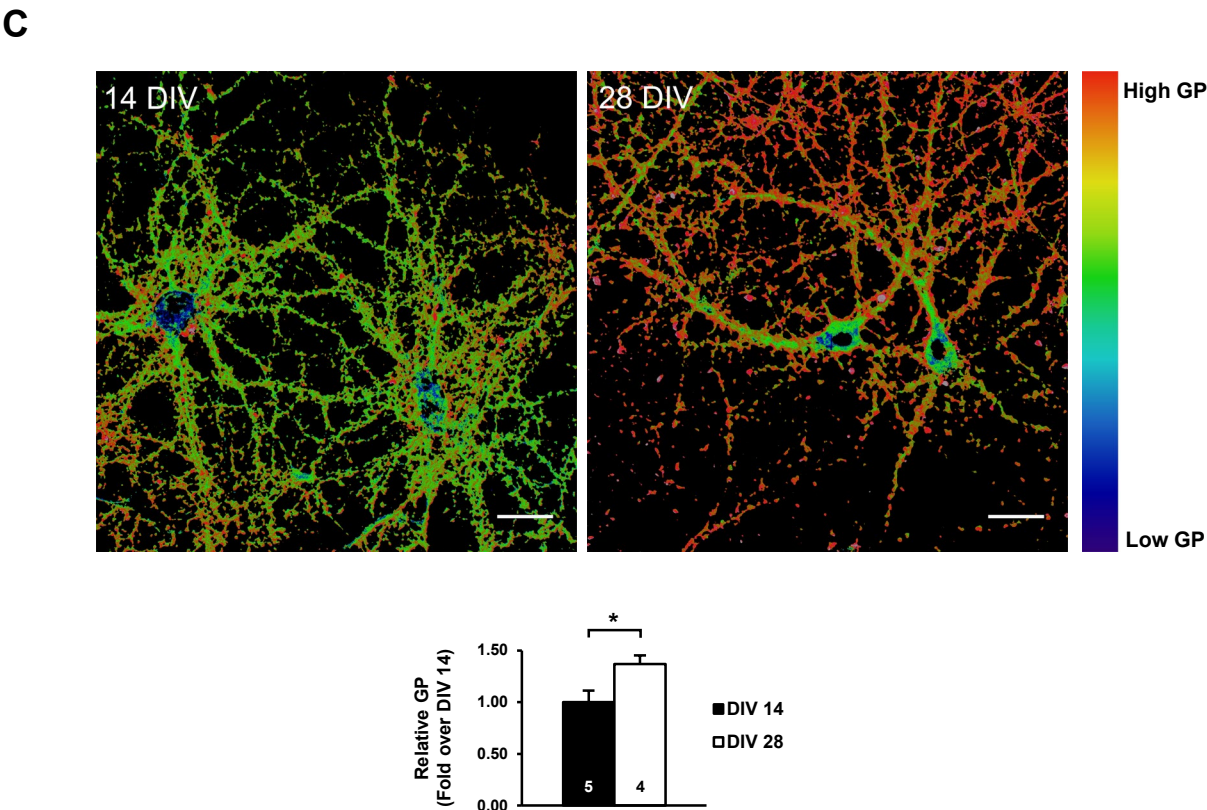

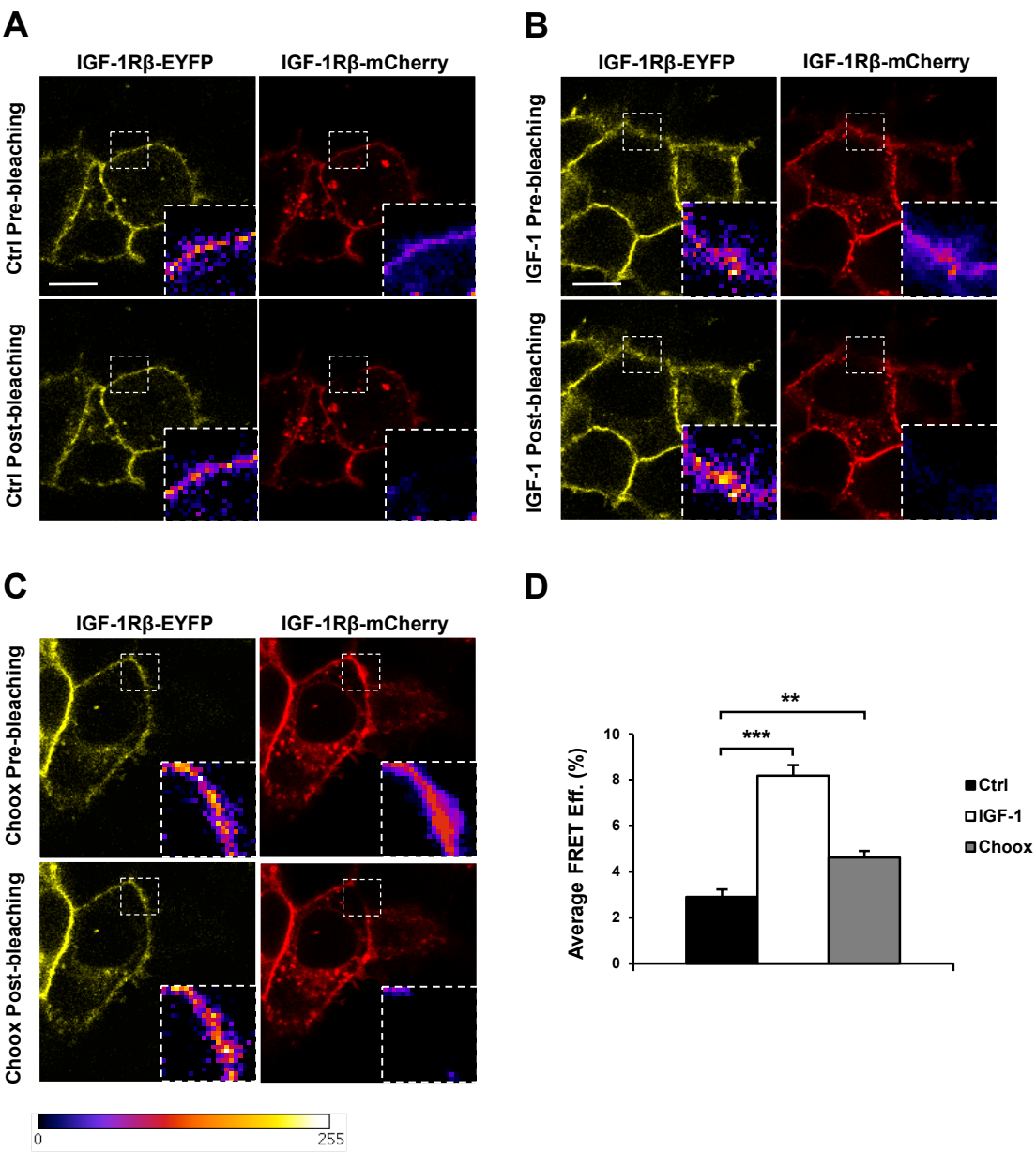

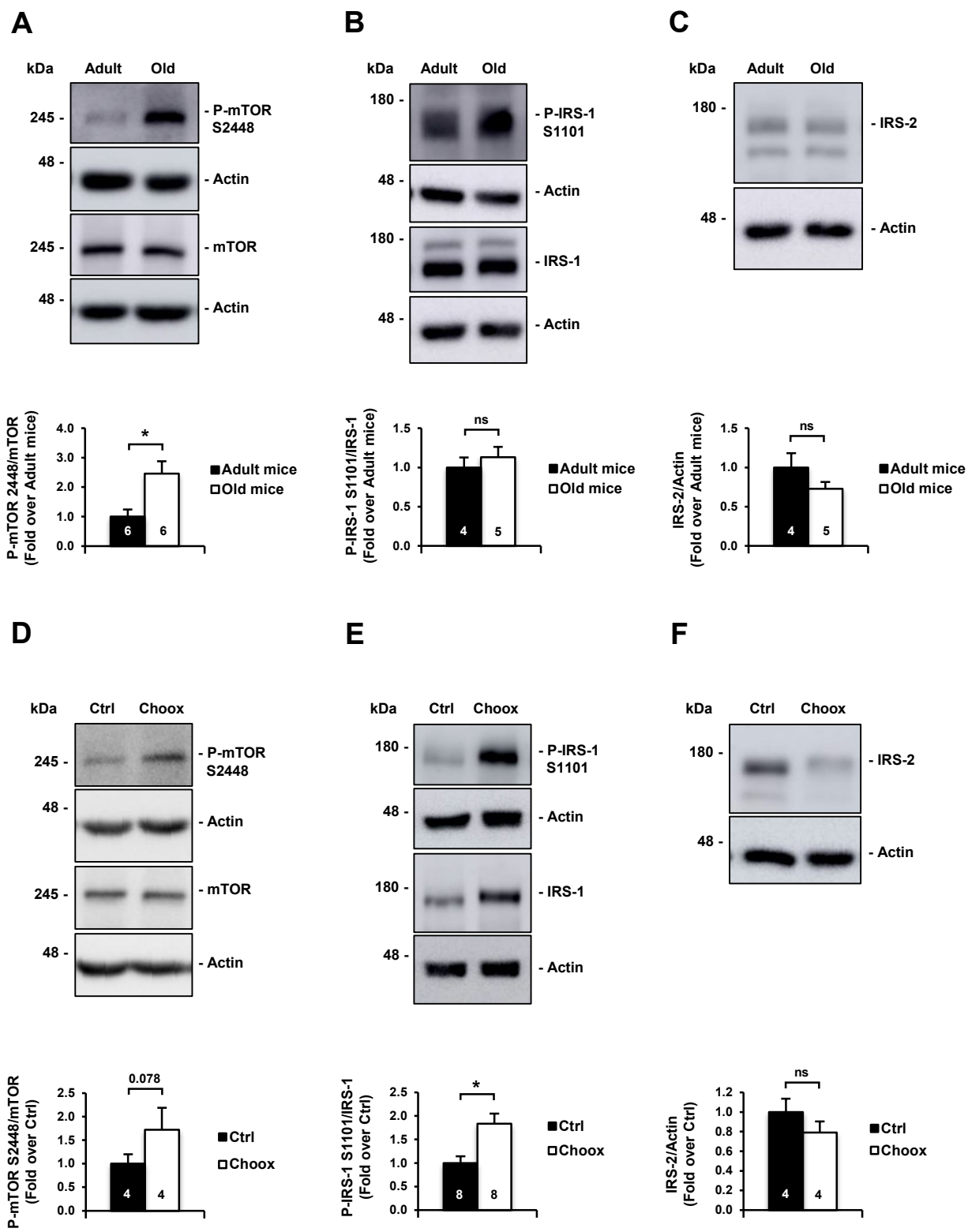

Supplement: Supplementary file 1 [file ACEL-18-e12932-s001.pdf]
